# Viperin is an important host restriction factor in control of Zika virus infection

**DOI:** 10.1038/s41598-017-04138-1

**Published:** 2017-06-30

**Authors:** Kylie H. Van der Hoek, Nicholas S. Eyre, Byron Shue, Onruedee Khantisitthiporn, Kittirat Glab-Ampi, Jillian M. Carr, Matthew J. Gartner, Lachlan A. Jolly, Paul Q. Thomas, Fatwa Adikusuma, Tanja Jankovic-Karasoulos, Claire T. Roberts, Karla J. Helbig, Michael R. Beard

**Affiliations:** 1 Molecular and Cellular Biology, Research Centre for Infectious Diseases, The University of Adelaide, Adelaide, SA 5005 Australia; 2 Adelaide Medical School and Robinson Research Institute, The University of Adelaide, Adelaide, SA 5005 Australia; 3 Centre for Cancer Biology, University of South Australia, Adelaide, SA 5000 Australia; 4 Microbiology and Infectious Diseases, School of Medicine, Flinders University, Bedford Park, SA 5042 Australia; 5 Department of Physiology, Anatomy and Microbiology, La Trobe University, Melbourne, Vic 3086 Australia

## Abstract

Zika virus (ZIKV) infection has emerged as a global health threat and infection of pregnant women causes intrauterine growth restriction, spontaneous abortion and microcephaly in newborns. Here we show using biologically relevant cells of neural and placental origin that following ZIKV infection, there is attenuation of the cellular innate response characterised by reduced expression of IFN-β and associated interferon stimulated genes (ISGs). One such ISG is viperin that has well documented antiviral activity against a wide range of viruses. Expression of viperin in cultured cells resulted in significant impairment of ZIKV replication, while MEFs derived from CRISPR/Cas9 derived viperin^−/−^ mice replicated ZIKV to higher titers compared to their WT counterparts. These results suggest that ZIKV can attenuate ISG expression to avoid the cellular antiviral innate response, thus allowing the virus to replicate unchecked. Moreover, we have identified that the ISG viperin has significant anti-ZIKV activity. Further understanding of how ZIKV perturbs the ISG response and the molecular mechanisms utilised by viperin to suppress ZIKV replication will aid in our understanding of ZIKV biology, pathogenesis and possible design of novel antiviral strategies.

## Introduction

Zika virus (ZIKV) is an arbovirus and a member of the *Flaviviridae* family that is a significant health threat on a global scale^1^. ZIKV gained global attention in 2007 when the first major outbreak was reported in Micronesia followed by smaller outbreaks in other pacific islands thereafter^2^. However, the largest outbreak to date was reported in 2015 in Brazil and was followed by widespread dissemination in Central and South America. While the majority of infections in humans are either asymptomatic or associated with fever, rash and conjunctivitis, the 2015 outbreak was associated with an alarming number of neurological and birth defects including microcephaly^3^. Evidence linking ZIKV to the above mentioned pathologies include ZIKV RNA present in the amniotic fluid and fetal and newborn brain tissue^4^. It is now well accepted that ZIKV can cross the placenta and subsequently infect neural progenitor cells of the fetus leading to significantly impaired brain development. ZIKV antigen has been identified in the placental chorionic villi from an infected mother who gave birth to a microcephalic infant and can also infect human placental trophoblasts and macrophages *in vitro* ^5, 6^. Moreover, recent mouse models have revealed that ZIKV infection can result in placental insufficiency, alterations in neural progenitor proliferation and fetal demise^7–10^. In addition to the classical mosquito transmission, ZIKV can be sexually transmitted via infected seminal fluid^11–13^. The growing association of ZIKV infection during pregnancy with serious neurological defects in newborns has resulted in ZIKV being declared as a public health emergency by the WHO.

The innate immune response to virus infection plays a crucial role in controlling viral infection in addition to shaping the adaptive immune response. This innate response is initiated by the recognition of genetic components expressed during viral replication, collectively termed pattern associated molecular patterns (PAMPs), by cellular sensors termed Pattern Recognition Receptors (PRRs)^14^. In the case of RNA viral infection, the best-characterized PRRs are the membrane bound toll-like receptors (TLRs) and the cytoplasmic RNA sensors RIG-I and MDA5^15^. Engagement of PRRs by viral PAMPs results in the activation of NF-κB, IRF-3 and IRF-7 and production of the type I interferons. Further amplification of the interferon (IFN) system occurs when IFN binds to the interferon receptor (IFNAR) and activates the Jak/Stat signaling cascade to ultimately drive expression of hundreds of interferon stimulated genes (ISGs). These ISGs inhibit viral replication and drive the inflammatory process in an attempt to limit viral replication. The importance of this system is exemplified by the fact that most viruses have evolved mechanisms to evade or inactivate the innate response^16^. As an example the *Flaviridae* members West Nile Virus (WNV) and dengue virus (DENV) target multiple sites following PRR activation and IFN signaling and most recently ZIKV has been shown to degrade STAT2 to inhibit ISG expression^17–19^. Furthermore the observation that mice deficient in the type I IFN receptor are more susceptible to ZIKV infection in comparison to WT mice highlights the importance of the innate response and ISG expression in controlling ZIKV infection^20–22^.

Although it is firmly established that the interferon response is an important determinant of host resistance, the antiviral mechanisms responsible for direct suppression of virus replication are less well understood. This, together with the observations that placental cells can resist ZIKV infection due to the actions of the type III IFN-λ and susceptibility of *Ifnar* ^−/−^ mice to ZIKV infection suggests a role for specific ISGs in control of ZIKV infection^21^. With this in mind we investigated the innate response to ZIKV infection and revealed that ZIKV infected cells of neural and placental origin fail to induce a robust ISG response. In particular we noted significant abrogation of expression of the ISG viperin that prompted us to investigate the antiviral role of viperin against ZIKV. We and others have demonstrated that the evolutionary conserved ISG viperin can inhibit the replication of a wide range of viruses that are responsible for significant disease in humans^23^. These include the *Flaviviridae* family members, DENV, TBEV, WNV and HCV^24–28^. Interestingly viperin exerts its antiviral effect by diverse mechanisms; for example viperin interacts with the HCV NS5A protein and the proviral host factor VAP-A, both of which are important in HCV replication, while in TBEV infection viperin restricts viral RNA replication dependent on the radical S-adenosyl-l-methionine (SAM) domain^25, 28, 29^. As for other *flaviviruses* we observed that expression of viperin in Huh-7 cells limited ZIKV replication, whereas viperin^−/−^ MEFs displayed heightened permissiveness to ZIKV infection. Taken together, this work suggests that the ability of ZIKV to impair innate immune recognition and specific ISG expression may be fundamental in enabling virus replication to proceed unchecked in specific cell types. Thus, viperin is a key ISG in controlling ZIKV infection. Understanding the ISGs that control ZIKV and other emerging viral infections is essential for defining mechanisms of viral pathogenesis and possible novel therapeutic strategies.

## Results

### The antiviral response is attenuated in placental and neural progenitor derived cell lines following ZIKV infection

**Figure 1:**
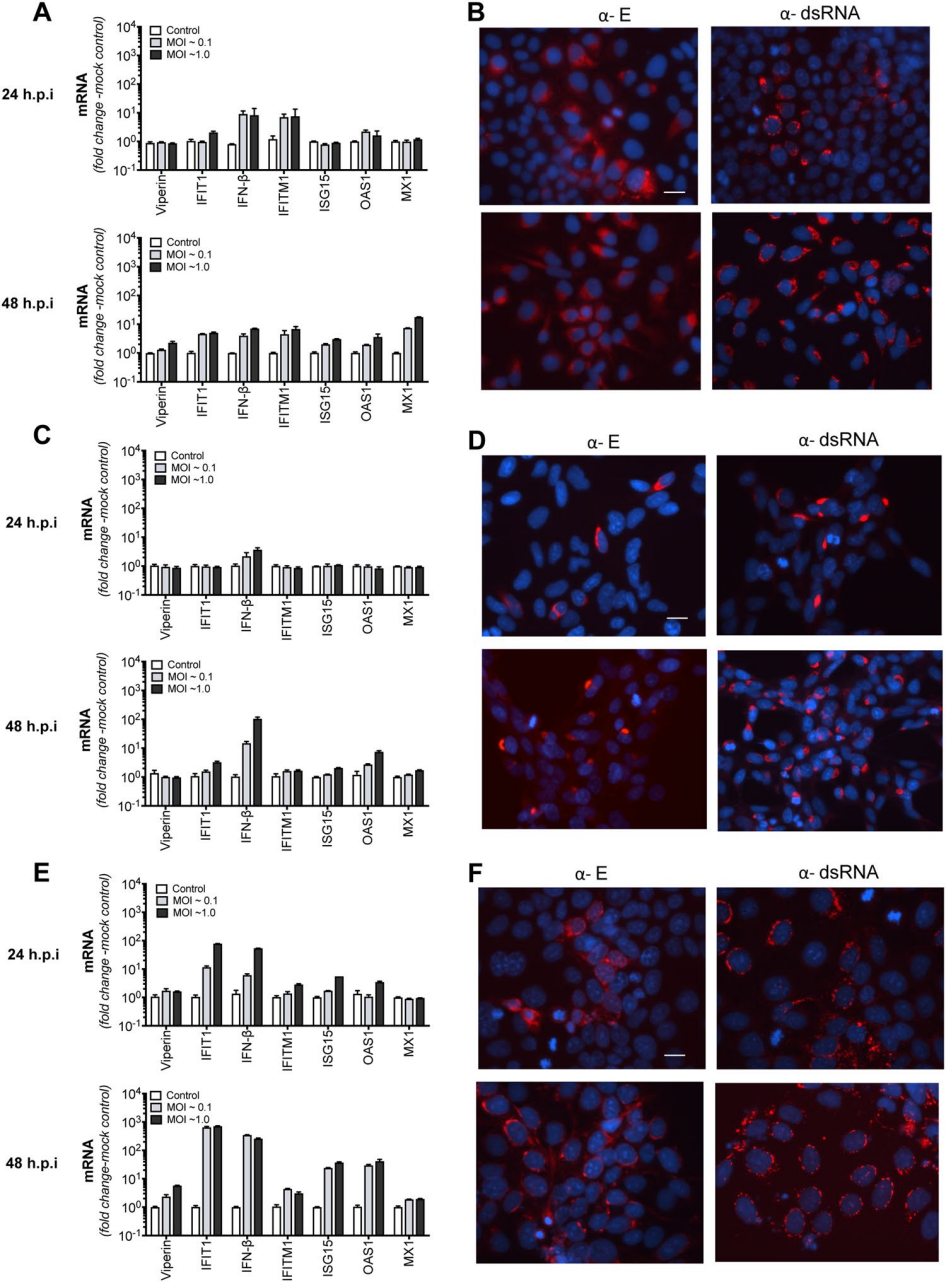
ZIKV infection results in an attenuated interferon-stimulated gene (ISG) response. (**A**,**C** and **E**) Huh-7, HTR8/SVNeo and Jeg3 cells, respectively were infected with ZIKV (MR766) at indicated multiplicity of infection (MOI). At 24 and 48 h.p.i total RNA was extracted and qRT-PCR was used to detect mRNA for IFN-β and the ISGs IFIT1, viperin, IFITM1, ISG15, OAS1 and Mx1. Data are normalised to the RPLPO housekeeping gene and expressed as a fold-change relative to mock-infected control (data are means + SD, n = 3). (**B**,**D** and **F**) Indirect immunofluorescence of corresponding ZIKV infection in Huh-7, HTR8/SVNeo and Jeg3 cells respectively. Cells were stained with the 4G2 antibody to detect ZIKV E antigen or 3G1 antibody to detect dsRNA (red) and DAPI DNA stain (blue). Scale bars represent 20 μm.

The cellular innate immune response to viral infection results in expression of the interferons and ISG expression that is critical to the establishment of an antiviral state. However, viruses can block this innate response and ZIKV is no exception with recent work showing that the ZIKV NS5 protein can degrade STAT2, a key transcription factor in the IFN signaling pathway^18, 19^. Host innate responses are often cell type specific and with this in mind, we investigated ISG expression in a range of cell types following ZIKV infection. Initially, we infected the liver derived cell line Huh-7 with ZIKV (MOI of 0.1 and 1.0) and assessed ISG mRNA expression by qRT-PCR and ZIKV infectivity using an antibody (4G2) that detects pan-flavivirus envelope including ZIKV. As previously described, Huh-7 cells were readily infected by ZIKV resulting in spreading infection that ultimately resulted in significant cytopathic effect (CPE) at 72 hr post-infection (h.p.i)^30^. We previously reported that infection of Huh-7 cells with the closely related flavivirus DENV results in significant ISG expression; however, this was not the case with ZIKV infection^24^. We quantitated the induction of IFIT1, viperin, IFN-β, IFITM1, ISG15, OAS1 and Mx1 mRNA by qRT-PCR at 24 and 48 h.p.i. Early in infection (24 h.p.i) cells were particularly unresponsive to ZIKV infection even though a significant proportion of the culture was infected (Fig. 1A,B). Even after establishment of infection (48 h.p.i) there was minimal expression of ISGs. In contrast, stimulation of these cells with the double-stranded RNA (dsRNA) mimic poly(I:C) resulted in robust induction of ISG expression (Supplementary Fig. S1), indicating that the weak response observed in ZIKV-infected cells cannot be attributed to defects in dsRNA sensing or signaling pathways in these cells.

Next we investigated ISG expression in a number of more physiologically relevant cell lines, namely the first trimester immortalised extravillious trophoblast line HTR8/SVNeo and JEG3 a trophoblast derived choriocarcinoma line^31^. Following ZIKV infection at MOI of 0.1 and 1.0, HTR8/SVNeo cells harbored productive viral replication as determined by immunolabeling of both E protein and dsRNA (Fig. 1D). Even in the face of significant viral replication at 24 and 48 h.p.i there was minimal or no induction of mRNA expression of our ISG panel and only a moderate increase in IFN-β mRNA at 48 h.p.i. (Fig. 1C). A similar pattern of ISG expression was seen following infection of cultures with ZIKV strain PRVABC59 confirming that this observation is not strain dependent (Supplementary Fig. S2). In contrast, JEG3 choriocarcinoma cells were more responsive to infection with increases, albeit moderate, in mRNA expression for IFIT1 and IFN-β at 24 and 48 h.p.i, however viperin expression was attenuated at both time points post ZIKV infection (Fig. 1E). As above, this attenuation of ISG mRNA expression was not a result of a defect in innate immune sensing or signaling as significant increases in ISG induction could be seen following stimulation with poly (I:C) in all cell-lines tested (Supplementary Fig. S1).

**Figure 2:**
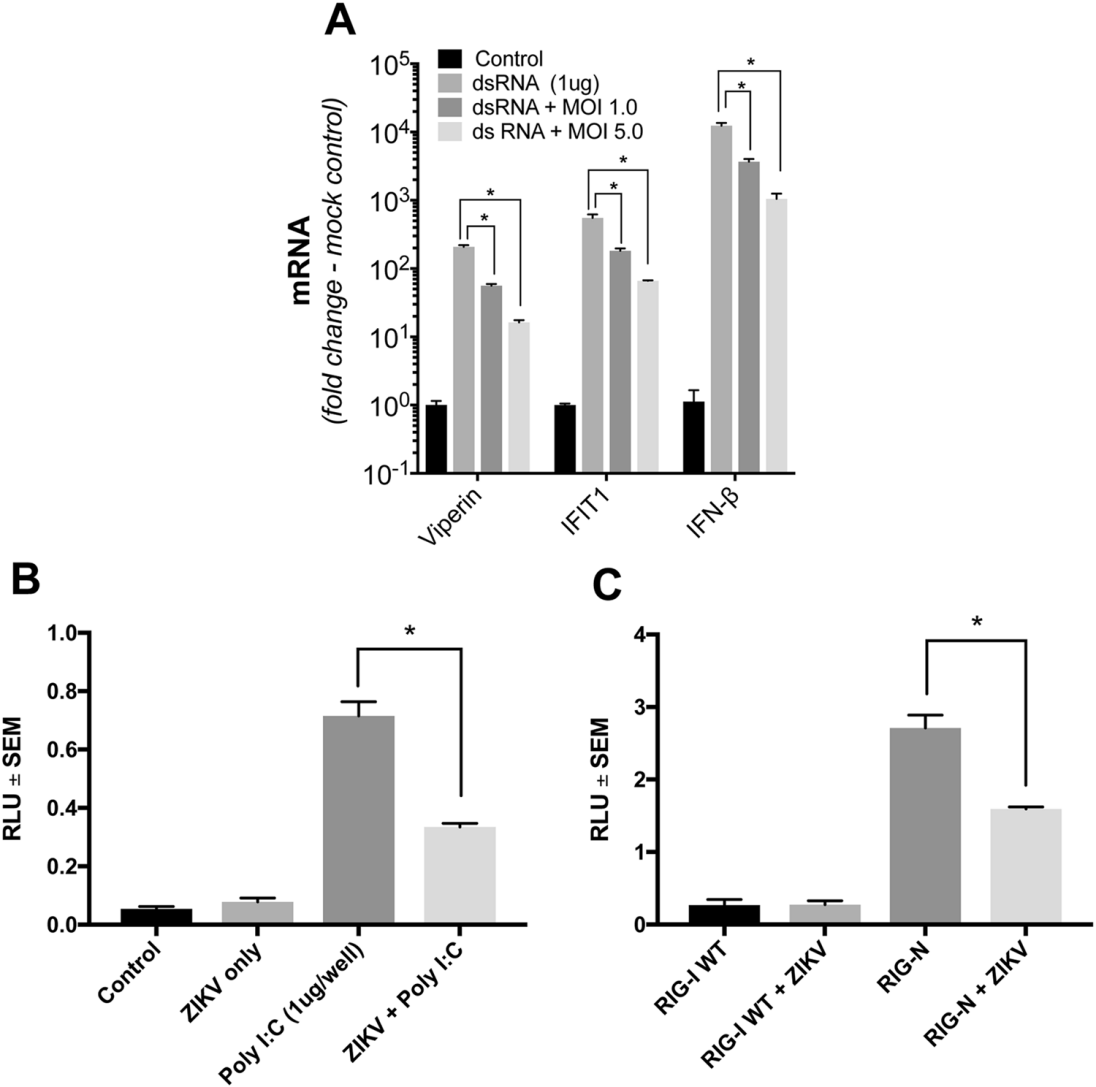
Poly I:C stimulated ISG expression and IFN-β promoter activity is attenuated in the presence of ZIKV infection. (**A**) Huh-7 cells were infected with ZIKV (MR766) at indicated MOIs and 16 h.p.i cells were transfected with polyI:C (1 μg). 24 hr later total RNA was extracted and qRT-PCR was used to detect mRNA for IFN-β and the ISGs IFIT1 and viperin. Data are normalised to the RPLPO housekeeping gene and expressed as a fold-change relative to mock-infected control (data are means + SD, n = 3, *P = 0.0003, Students t-test). (**B**) Huh-7 cells were transfected with a luciferase reporter plasmid driven by the IFN-β promoter followed by infection with ZIKV (MR766, MOI 5). 16 h.p.i cells were transfected with polyI:C and 24 hr post transfection cell lysates were harvested for luciferase assay. Data are normalised to transfection with TK-Renilla plasmid and expressed as a relative light units compared to mock-infected control (data are means + SEM, n = 3, *P = 0.003, Students t-test). (**C**) Huh-7 cells were transfected with either a plasmid expressing WT RIG-I or a constitutively active RIG-I (RIG-N), together with a luciferase reporter plasmid driven by the IFN-β promoter followed by infection with ZIKV (MR766, MOI 5). 24 h.p.i cell lysates were harvested for luciferase assay. Data are normalised to transfection with TK-Renilla plasmid and expressed as a relative light units compared to uninfected cells (data are means + SEM, n = 3, *P = 0.002, Students t-test).

Our results suggest that while ZIKV may induce a cellular innate response, the early response may be actively attenuated. To investigate this further we quantified viperin, IFIT1 and IFN-β mRNA following polyI:C stimulation of Huh-7 cells in the presence or absence of ZIKV infection. As expected, mRNA for viperin, IFIT1 and IFN-β significantly increased following polyI:C stimulation, however this was significantly attenuated in the presence of ZIKV infection (Fig. 2A). Consistent with the notion that ZIKV can actively suppress early ISG expression we also demonstrate that ZIKV can attenuate the IFN-β promoter in response to both polyI:C and expression of constitutively active RIG-I (RIG-N) (Fig. 2B,C). RIG-I PAMP recognition ultimately induces IRF3 phosphorylation and nuclear translocation and collectively these results suggest that ZIKV can actively reduce RIG-I sensing of ZIKV RNA and downstream ISG expression.

**Figure 3:**
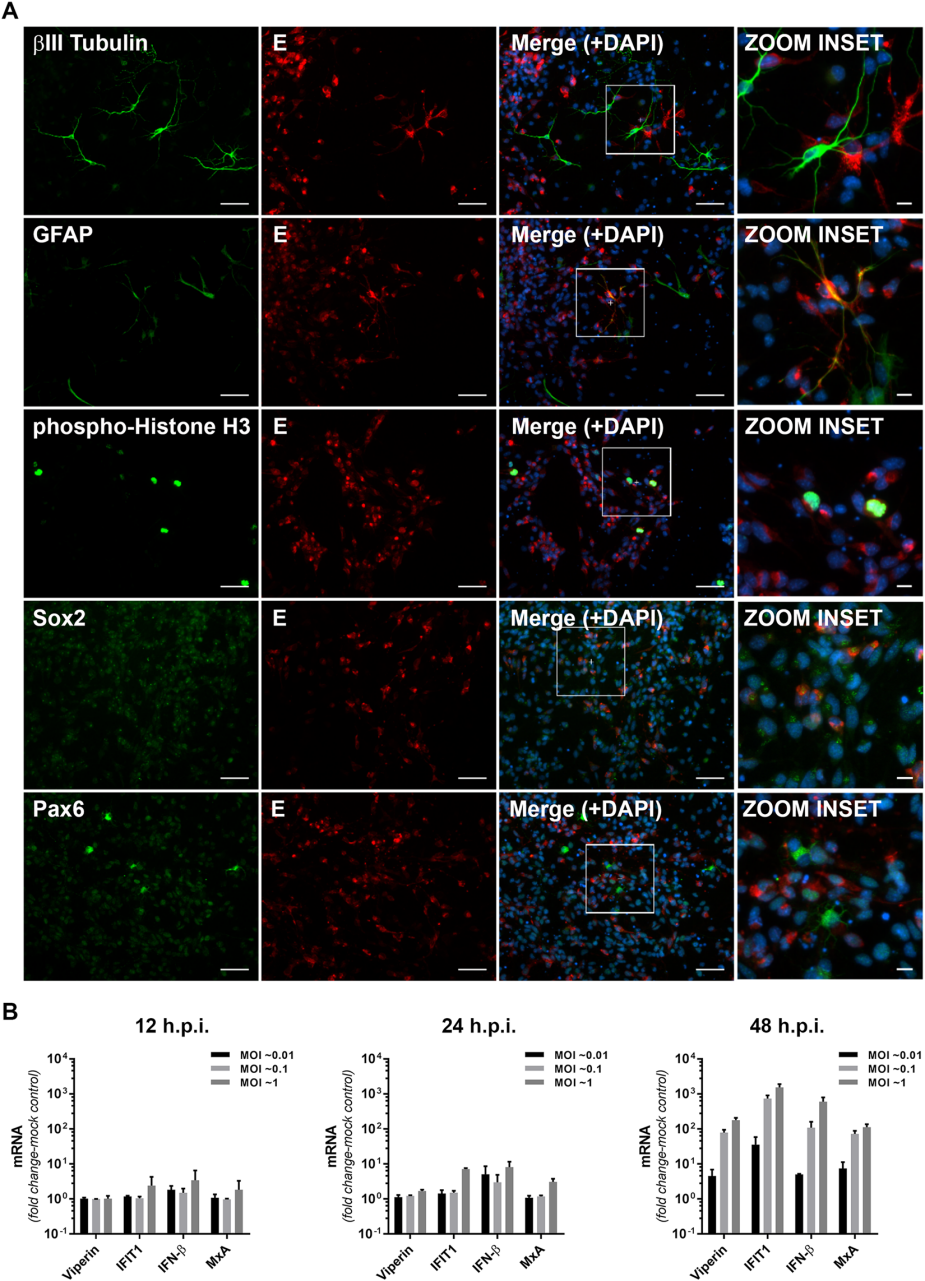
Mouse neural progenitor cells (NPCs) are susceptible to ZIKV infection and display delayed interferon-stimulated gene (ISG) expression responses. (**A**) Immunofluorescence analysis of ZIKV infection in neural cell subpopulations within NPC cultures. NPCs were infected with ZIKV (MR766, MOI: 0.1) for 48 h prior to fixation and staining of the ZIKV E protein (red) in combination with βIII tubulin (neurons), GFAP (astrocytes), phospho-Histone H3 (mitotic cells), Sox2 (apical progenitor cells) or Pax6 (radial glia) markers of neural cell sub-populations (green). Nuclei were counterstained with DAPI (blue). Scale bars are 100 μm for main images and 10 μm for ‘zoom insets’. (**B**) Mouse neural progenitor cells were infected with ZIKV (MR766) at the indicated MOI before extraction of total cell RNA at 12–48 h.p.i and analysis of ISG expression by qRT-PCR. Data are normalised to the RPLPO housekeeping gene and expressed as a fold-change relative to mock-infected control (data are means + SD, n = 3).

ZIKV has been shown to infect both human and mouse neural progenitor cells (mNPCs) resulting in cell-cycle arrest and apoptosis thus providing a direct potential link between ZIKV infection and microcephaly^8, 9, 30, 32^. We therefore investigated innate responses to ZIKV (MR766 strain) infection, in NPCs derived from mouse E14-18 cortices. To investigate which cells were infected by ZIKV, cultures were immunostained using Sox2 and Pax6 as markers of apical progenitor cells and radial glial cells respectively, GFAP to identify astrocytes, βIII-Tubulin to identify neurons and histone H3 to identify mitotic cells. ZIKV infection was noted as early as 12 h.p.i with a significant number of cells in the culture positive for ZIKV E antigen at 48 h.p.i (Fig. 3A). Consistent with previous reports *in vitro* and *in vivo*, ZIKV-infected cells were positive for Sox2, Pax6 and GFAP suggesting the potential for ZIKV to infect NPCs and, to a lesser extent, astrocytes in the developing brain (Fig. 3A)^9, 30, 33, 34^. Next we investigated IFN-β and ISG expression at various time points post ZIKV infection. Consistent with our observations above there was a delayed and attenuated response to ZIKV infection with IFN-β and ISG responses not seen until 48 h.p.i (Fig. 3B). Given that this culture is a mixed population of cells, it is unclear at this stage if the ISG response is derived from ZIKV infected cells or from uninfected bystander cells that may be responding to low levels of IFN-β produced from infected cells. We also infected mNPC cultures with ZIKV strain PRVABC59 and observed similar innate immune responses to infection with MR766, suggesting that the innate response noted above is not strain dependent (Supplementary Fig. S3). Further experiments are required to determine the cell types responsible for ISG expression in NPC cultures. Nevertheless, these results suggest that primary neural progenitor cells also have a delayed innate response to ZIKV infection.

### ZIKV infects and induces an antiviral response in monocyte-derived macrophages

**Figure 4:**
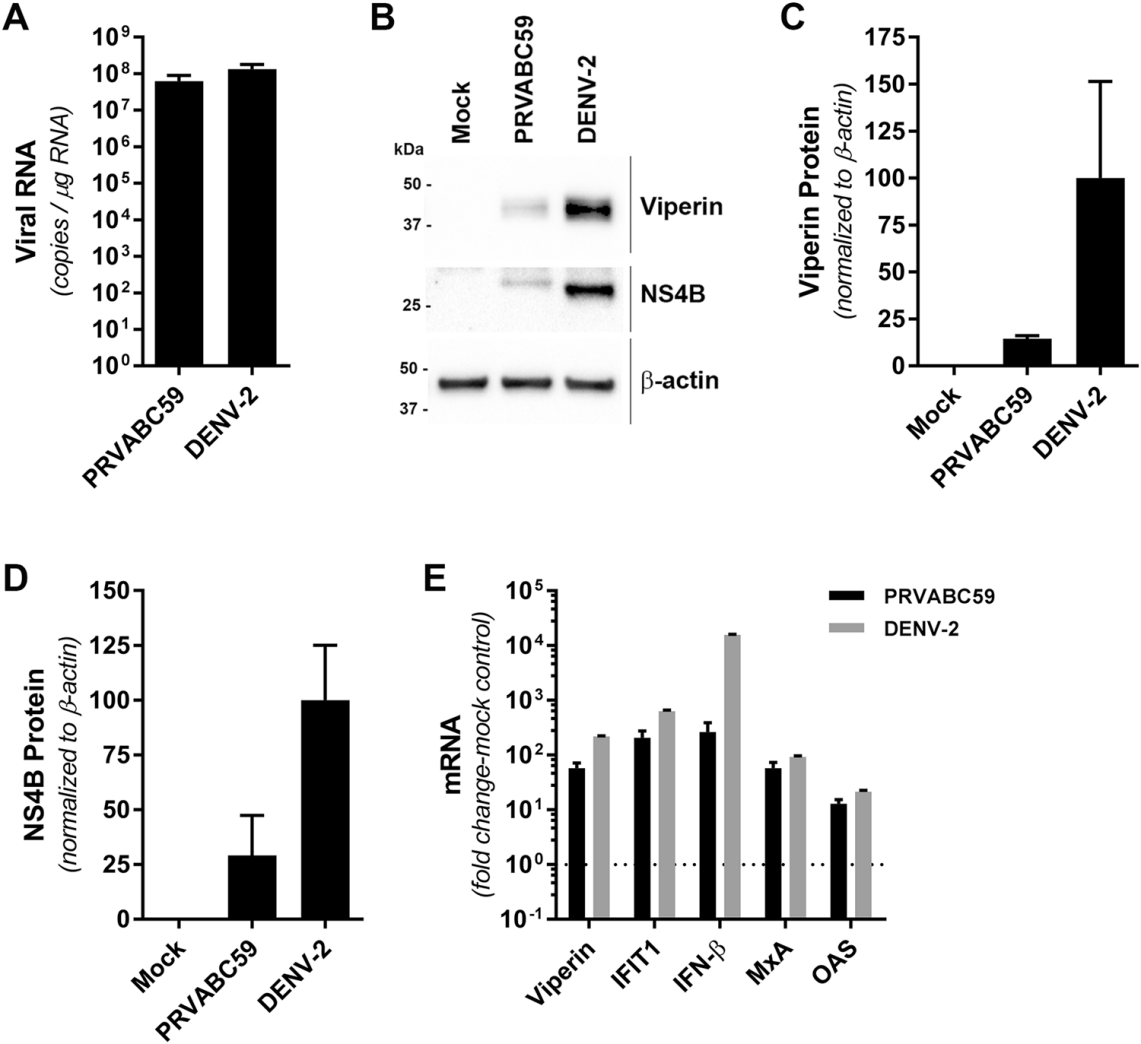
Human monocyte-derived macrophages are moderately susceptible and responsive to ZIKV infection. Human monocyte-derived macrophages were infected with ZIKV (PRVABC59) or DENV-2 (Mon601) for 48 h (MOI = 1) prior to analysis of viral replication and ISG expression by qRT-PCR and Western blotting in parallel. (**A**) ZIKV and DENV-2 RNA levels were determined by qRT-PCR using plasmid DNA standard curves. Data are expressed as viral RNA copy numbers per μg of total cell RNA (means + SD, n = 3). (**B**) Parallel cultures were subjected to immunoblotting analysis of viperin and NS4B expression, with β-actin serving as a loading control. (**C**,**D**) Quantitation of Viperin and NS4B protein levels, normalized to β-actin (means + SD, n = 3). (**E**) ISG mRNA levels were determined by qRT-PCR. Data are normalised to the RPLPO housekeeping gene and expressed as a fold-change relative to mock-infected controls (means + SD, n = 3).

ZIKV has been shown to infect human placental macrophages (Hofbauer cells [HCs])^5^ and given that the closely related flavivirus, DENV targets primary monocyte-derived macrophages (MDM) *in vivo*, we also investigated if MDMs were also permissive to ZIKV infection and the associated antiviral response. In this instance we used a low cell culture passaged ZIKV strain PRVABC59, isolated from the sera of an infected patient in Puerto Rico in 2015 that is closely related to the epidemic strains circulating in the Americas that have been linked to *in utero* ZIKV infection. We demonstrate that MDMs are permissive to productive ZIKV infection as determined by both qRT-PCR and immunoblot for detection of NS4B (Fig. 4A,B,C,D). We also evaluated the antiviral potential of MDMs infected with ZIKV. MDMs were infected with ZIKV (MOI 1.0) and 48 h.p.i mRNA for viperin, IFIT1, IFN-β, MxA and OAS were quantified (Fig. 4E). For comparison we also infected MDMs with DENV (MOI 1.0), given that these cells are a well-validated target cell *in vivo*. We observed increased expression of transcripts for viperin, IFIT1, IFN-β, MxA and OAS at 48 h.p.i for both ZIKV and DENV. However, transcript levels were significantly more elevated in DENV infection (Fig. 4E), although this may be a result of more efficient DENV replication as evident by qRT-PCR and immunoblot analysis (complete unprocessed immunoblots can be seen in Supplementary Fig. S4) of DENV RNA and NS4B expression respectively (Fig. 4A,B). Most notable was the significantly elevated expression of IFN-β in DENV, compared to ZIKV infected MDMs that may in turn drive higher viperin and IFIT1 mRNA and viperin protein expression. Collectively these results show that MDMs are susceptible to ZIKV infection and respond through activation of antiviral signaling pathways.

### The antiviral ISG viperin modulates ZIKV replication

**Figure 5:**
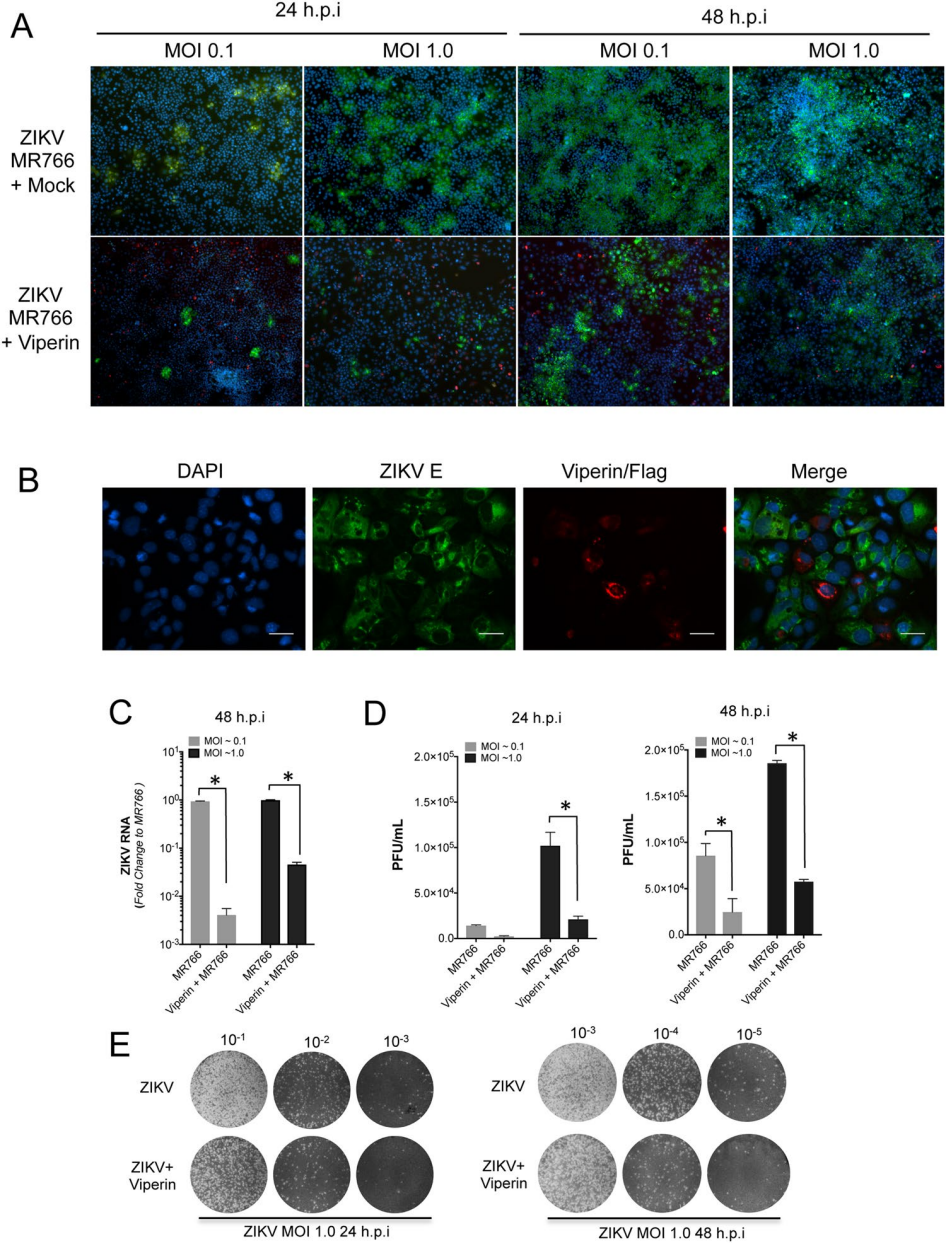
Viperin expression inhibits ZIKV infection in Huh7 cells. (**A**,**B**) Huh7 cells were mock transfected or transfected with a plasmid expressing viperin-FLAG. 16 hr post transfection, cells were infected with ZIKV (MR766) at the indicated MOI. Cells were fixed at the indicated time points and stained for ZIKV E antigen (green) in combination with viperin-FLAG (red). Nuclei were counterstained with DAPI (blue). Panel A 4x magnification. Scale bar on high-power images in (**B**) represents 50 μm. (**C**) RNA was extracted from ZIKV (MR766) infected Huh7 cells and ZIKV RNA levels analysed by qRT-PCR. Data is normalised to the RPLPO housekeeping gene and expressed as a fold-change relative to mock-infected controls (data are means + SD, n = 3, *p ≤ 0.0001, Two-Way Anova). (**D**,**E**) Supernatants from ZIKV (MR766) infected Huh7 cells were collected and plaque assay performed using Vero cells. Plaques were counted and averaged to deduce plaque forming units and subsequently ZIKV infectivity load. A representative plaque assay at 24 and 48 h.p.i is presented. (*p ≤ 0.03, Two-Way Anova).

Our results above revealing attenuated viperin mRNA induction following ZIKV infection prompted us to investigate the antiviral nature of viperin against ZIKV. We and others have shown that viperin restricts replication of the related *flaviviridae* family members, HCV, DENV TBEV and WNV^24, 25, 27–29^. To investigate the antiviral potential of viperin against ZIKV we transfected a plasmid that drives viperin expression into Huh-7 cells and 16 hr post transfection cells were infected with ZIKV strain MR766 at an MOI of 0.1 and 1.0 and analysis of viral replication was assessed at 24 and 48 h.p.i. Consistent with previous observations with other flaviviruses, viperin restricted replication of ZIKV MR766. In Huh-7 cells expressing viperin, compared to control cells there was a significant decrease in the proportion of ZIKV positive cells at both 24 and 48 h.p.i as determined by immunofluorescence microscopy to detect the ZIKV E protein (Fig. 5A). Even though approximately 30–40% of cells expressed viperin we could rarely detect cells positive for both viperin and ZIKV (Fig. 5B). Quantitative analysis of ZIKV RNA and release of infectious virus by qRT-PCR and plaque assay concurred with the microscopy analysis showing that compared to mock transfection, Huh-7 cells expressing viperin displayed a significant decrease in ZIKV RNA and production of infectious ZIKV 24 and 48 h.p.i. (Fig. 5C,D,E). Collectively these results indicate that the ISG viperin has significant antiviral activity against ZIKV.

**Figure 6:**
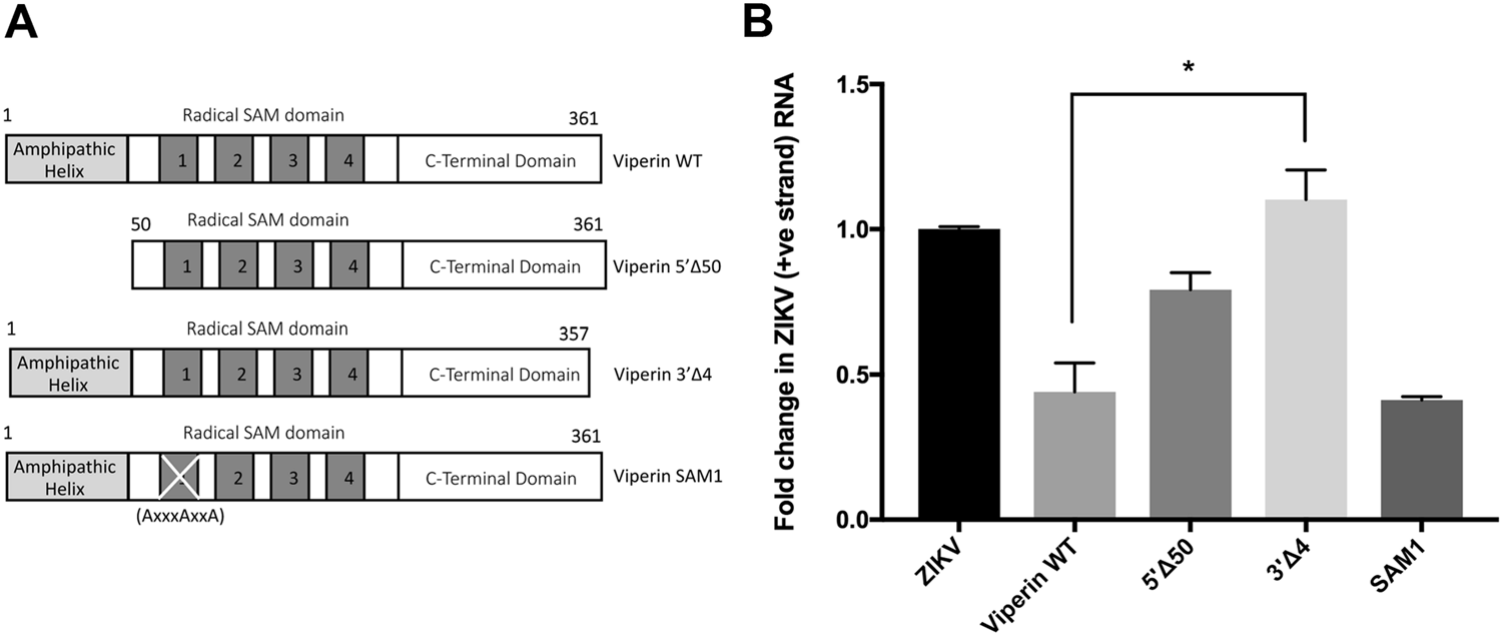
The C-terminal region of viperin is responsible for anti-ZIKV activity. (**A**) Schematic of Viperin WT and associated mutants used to transfect Huh-7 cells to assess the domains responsible for viperin’s anti-ZIKV activity. (**B**) Huh-7 cells were transfected to express WT or viperin mutants and at 24 hr infected with ZIKV (MR766, MOI = 1). RNA was extracted and ZIKV RNA quantitated by qRT-PCR. Results were normalised against control RPLPO mRNA levels and expressed as fold change (data are means + SD, n = 3, *p ≤ 0.025, Students t-test).

Next we investigated the anti-viral potential of viperin against ZIKV using a panel of viperin mutants that have previously been used to investigate viperin’s antiviral activity against the closely related flavivirus, DENV^24^. Huh-7 cells were transfected with the WT and mutant viperin constructs after which cells were infected with ZIKV and ZIKV RNA quantified by qRT-PCR (Fig. 6). Consistent with our previous results with DENV, the radical SAM domain mutant (SAM1) and the amino-terminal deletion mutant (5′Δ50) retain antiviral activity while the carboxy-terminal deletion mutant (3′Δ4) abolished viperin’s anti-ZIKV activity (Fig. 6). These results highlight the importance of the carboxy-terminal domain of viperin for anti-viral activity against ZIKV.

### CRISPR/Cas9-derived Viperin null MEFs result in increased ZIKV infection

Murine viperin has been shown to limit replication of a number of viruses *in vivo* ^27, 35^. To assess the impact of viperin deletion on ZIKV infection, we generated viperin null mice using CRISPR/Cas9 genome editing. Our approach used a gRNA to create indels in the ORF of exon I, thus generating frameshift null alleles. All 16-founder mice generated from CRISPR/Cas9 injection contained indels (mostly deletions) around the expected position within the *viperin* coding sequence as shown by Sanger sequencing, suggesting highly efficient editing of CRISPR/Cas9 system in our experiment. A 32 bp deletion derived from founder #67 and a 113 bp deletion derived from founder #72 were selected for further analysis (referred to hereafter as *Vip* ^−/−^ *del32* and *Vip* ^−/−^ *del113 respectively*). In both lines of mutant mice, these deletions are predicted to generate a premature stop codon within the first viperin exon. Viperin immunoblot analysis of the isolated MEFs from both lines of mice following IFN-α stimulation revealed an absence of viperin expression (results not shown), indicating successful disruption of the *viperin* locus in the viperin KO lines.

**Figure 7:**
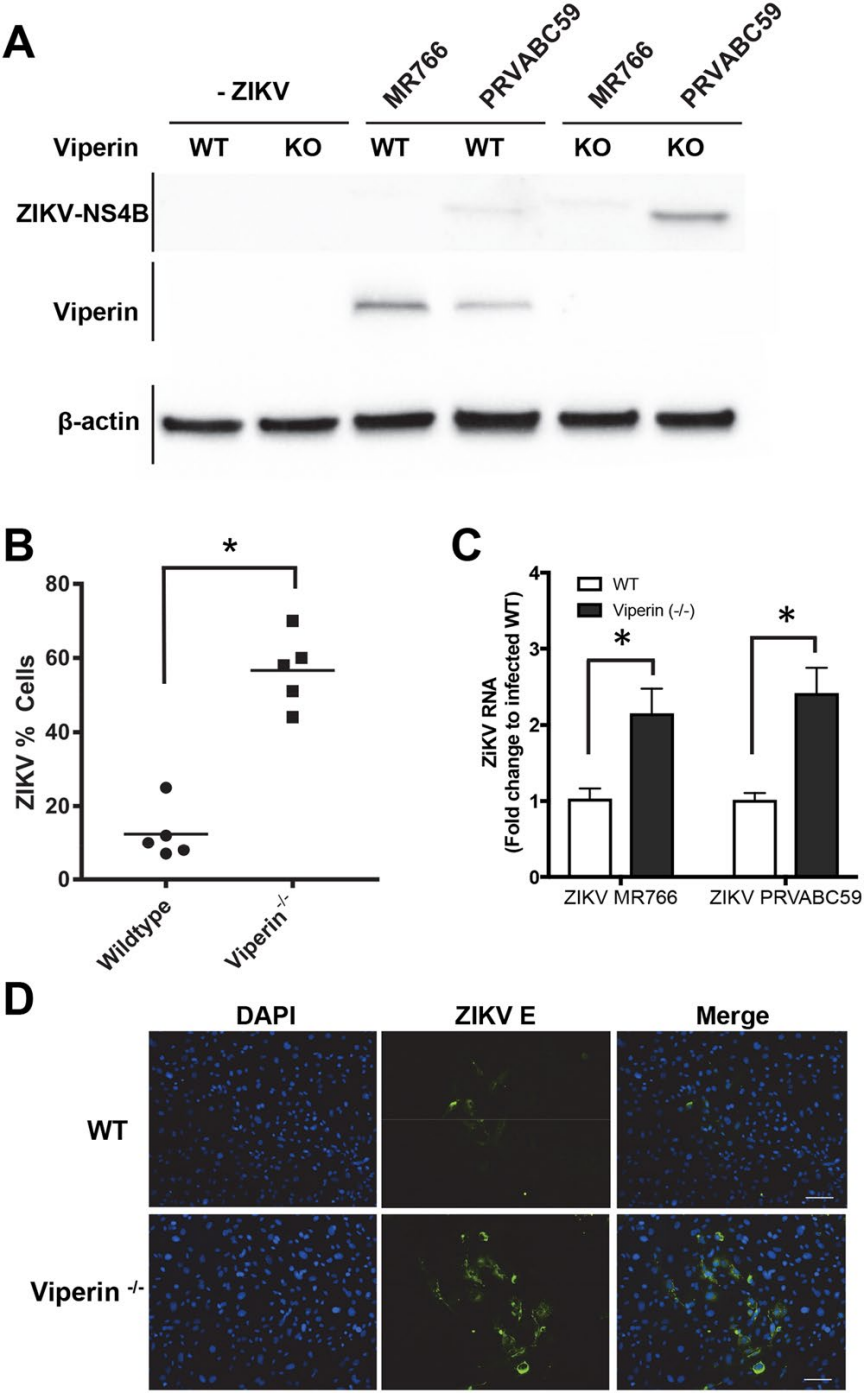
Viperin knockout mouse embryonic fibroblasts (MEF) are more susceptible to ZIKV infection. (**A**) MEFs derived from either WT or CRISPR/Cas9 viperin Ko (*Vip* ^−/−^ *del32*) mice were infected with ZIKV (MR766 or PRVABC59 at MOI ~1) for 48 hours prior to immunoblot analysis of Viperin and ZIKV-NS4B expression, with β-actin serving as a loading control. (**B**) MEFs from WT and viperin KO (*Vip* ^−/−^ *del32*) were infected with ZIKV (MR766) at an MOI of 1.0 and at 48 h.p.i the number of ZIKV positive cells (E antigen) was counted using Nikon ImageQuant software. (*p ≤ 0.0001, Students t-test). (**C**) RNA was extracted from ZIKV (MR766) infected WT and *Vip* ^−/−^ *del32* MEFs 48 h.p.i and ZIKV RNA levels analysed by qRT-PCR. Data is normalised to the RPLPO housekeeping gene and expressed as a fold-change relative to mock-infected control (data are means + SD, n = 3). (*p ≤ 0.02, Two-Way Anova). (**D**) Indirect immunofluorescence of corresponding ZIKV infection of WT and *Vip* ^−/−^ *del32* MEFs. Cells were stained with the 4G2 antibody to detect ZIKV E antigen (green) and DAPI DNA stain (blue). Scale bar represents 100 μm.

Next we investigated the role of viperin in controlling ZIKV replication in *Vip* ^−/−^ *del32* mouse embryonic fibroblasts (MEFs) following ZIKV infection. Infection of WT and *Vip* ^−/−^ *del32* MEFs with either ZIKV strains MR766 or PRVABC59 revealed that at 48 h.p.i viperin expression was induced in the WT MEFs but not the *Vip* ^−/−^ *del32* MEFs indicating that the innate cellular response to ZIKV infection is activated and results in viperin expression (Fig. 7A). Moreover, in the *Vip* ^−/−^ *del32* MEFs there was a significant increase in ZIKV NS4B protein compared to WT MEFs suggesting that endogenous viperin can restrict ZIKV replication (complete unprocessed immunoblots can be seen in Supplementary Fig. S5). This was further confirmed by immunofluorescence analysis of infected cultures that revealed an increased proportion of ZIKV positive cells in *Vip* ^−/−^ *del32* MEFs compared to WT MEFs (Fig. 7B,D). Furthermore quantification of ZIKV RNA by qRT-PCR analysis from infected cultures of WT and *Vip* ^−/−^ *del32* MEFs revealed a significant increase in ZIKV RNA in the *Vip* ^−/−^ *del32* MEFs at 48 h.p.i. (Fig. 7C). It should be noted that this represents experiments using MEFs derived from *Vip* ^−/−^ *del32*, however similar results were also obtained with the independent *Vip* ^−/−^ *del113* line. These results reveal that endogenously expressed viperin can limit ZIKV infection of MEFs and together with our transfection studies above indicate that viperin is a host innate restriction factor for ZIKV infection.

## Discussion

The host innate response to viral infection is critical in controlling viral replication through the induction of a type I interferon response and ISG expression which serves to limit viral replication and accelerate the inflammatory process^36^. Recent studies using mice with a defect in the type I interferon signaling axis such as *Ifnar* or *Irf3/5/7* knockout mice develop neurological disease and increased viral replication in the brain, spinal cord and testes following ZIKV infection^7^. Moreover, *in utero* infection of *Ifnar* knock out mice early in pregnancy resulted in ZIKV infection of the placenta, and brain causing intrauterine growth restriction and spontaneous abortion^37^. Further evidence for the importance of the type-I response comes from recent work showing that like DENV, the NS5 protein of ZIKV abrogates IFN signaling through NS5 protein binding to STAT2 inducing its proteosomal degradation^18, 19^. These results highlight the importance of the type I interferon response in controlling ZIKV replication and pathogenesis, but the molecular mechanisms that underpin this restriction are not well understood. However, as for other viral infections, host control is most likely a combination of antiviral ISG expression, initiation and exacerbation of an inflammatory response and maturation of the adaptive antiviral response.

With this in mind we investigated the role that ZIKV infection has in induction of antiviral gene expression by measuring the induction of IFN-β and a panel of ISGs (viperin, IFIT1, IFITM1, ISG15, OAS and Mx1) at various time points following infection using physiologically relevant cell lines of placental and neurological origin. Consistent with other *flavivirus* infections we expected to see an early response to infection that is mediated independently of the synthesis of IFN-β. However, even at 24 h.p.i significant induction of IFN-β or ISGs in Huh-7 cells, HTR8/SVNeo (first trimester trophoblastic line) or murine neural progenitor cell cultures was not detected even though there was significant ZIKV replication. This attenuation of ISG expression seems to be an active process as polyI:C stimulation of ZIKV infected Huh-7 cells resulted in a significant reduction in ISG expression (Fig. 2A). Interestingly the JEG3 choriocarcinoma cell line, displayed a robust ISG response to infection suggesting that the ISG response may be determined by a combination of viral suppression of the innate response and cell intrinsic factors. While it has been reported that ZIKV can inhibit innate signaling post engagement of the type I IFN receptor, as discussed above, the lack of an early response is likely indicative of interferon independent suppression^18, 19^. IFN-β transcription is coordinately regulated by IRF3/7 and NF-κB and numerous ISGs such as IFIT1 and viperin can also be regulated independently of IFN by IRF3^38, 39^. Accordingly, the lack of expression of IFN-β and ISGs suggests that ZIKV may impact innate immune signaling upstream of IFN signaling. This is not inconceivable as DENV NS2B/3 can block the kinase activity of IKKε and cleave human STING while NS2A and NS4B can inhibit the autophosphorylation of TBK1 thereby inhibiting IRF3 phosphorylation^40–42^. Moreover, it was recently reported that ZIKV can suppress reporter expression from the IFN-β, IRF3 and NF-κB promoters^19^. With this in mind we confirmed that ZIKV can suppress IFN-β promoter activity in response to polyI:C and expression of constitutively active RIG-I (RIG-N) indicating that the blockade occurs post engagement of RIG-I (Fig. 2B,C). Clearly more work is required to determine the molecular mechanisms that underpin ZIKV mediated suppression of the early innate immune response upstream of IFN signaling.

We also investigated ZIKV infection and its ability to induce IFN-β and ISGs in primary MDMs a defined target cell type for the related DENV. ZIKV has been previously shown to infect and replicate in primary human placental macrophages (HC: Hofbauer cells) resulting in production of IFN-α and associated cytokines and ISGs^5^. Similarily, in MDMs we demonstrate significant expression of IFN-β, IFIT1, viperin, MxA and OAS mRNA and viperin protein expression at 48 hpi. Interestingly, even though both DENV and ZIKV infection of MDMs resulted in a similar number of infected cells, as determined by qRT-PCR, DENV infection resulted in greater levels of DENV NS4B protein and significantly higher viperin expression, suggesting that DENV RNA may be a more potent inducer of the innate response or that ZIKV can suppress innate immune activation. However, this is complicated by the possibility that DENV infection of MDMs may be more efficient than that of ZIKV and further experiments are required to dissect this difference. Interestingly, it should be noted that the observed innate response is the result of a relatively small proportion of MDMs directly infected with ZIKV (and for DENV), an observation that was also reported in the infection of HC cells (0.6–6.3% infection rate) suggesting that ISG expression may be largely derived and amplified from uninfected bystander cells in response to type I IFN production that would contribute to making bystander cells refractory to infection.

Activation of the innate immune response following RNA viral infection results in the production of type I interferons and the production of hundreds of IGS. While antiviral properties have been assigned to a number of these ISGs, the role of many of these ISGs remain undefined^36^. Given the growing list of ISGs with antiviral action against RNA viruses, it is likely that multiple ISGs act in a coordinated response in an attempt to limit viral infection. As an example it has recently been reported that the IFITM family of ISGs can limit ZIKV as it also does for a number of other RNA viruses including HCV^43, 44^. Interestingly, we noted during our investigations that the antiviral ISG viperin was only weakly induced following ZIKV infection of cells of placental and neural origin, and prompted us to investigate the antiviral potential of viperin against ZIKV. Viperin is strongly induced following a number of viral infections and has been shown by many laboratories including ours to be antiviral against HCV, DENV, WNV, TBEV, HIV, CHIV and HCMV (reviewed in ref. 23). We now show in this study that ZIKV can also be added to the list of viruses that can be restricted by viperin. Using a similar based approach to our previous studies in which we transiently expressed viperin and coupled with MEFs isolated from CRISPR/Cas9 derived viperin null mice we clearly demonstrate that viperin can limit ZIKV infection. Together these data clearly demonstrate that viperin is a potent host restriction factor of ZIKV replication.

The molecular mechanism(s) that underpins viperin-mediated viral restriction varies between viruses and even between viruses of the same family. Viperin restriction of HIV and influenza A seems to be mediated at the stage of viral egress in which viperin can disrupt lipid raft formation and membrane fluidity via dysregulation of cholesterol biosynthesis by inhibition of farnesyl diphosphate synthase a key enzyme in cholesterol and sterol biosynthesis^45, 46^. In contrast restriction of HCV and DENV (both *Flaviviridae* family members) is at the level of viral RNA replication. Here, viperin demonstrates antiviral restriction against a HCV replicon and interacts with both HCV NS5A and with the cellular pro-viral host factor VAP-A that are both viral replication complex components essential to HCV replication^25, 29^. Furthermore viperin co-localized and co-precipitated with dsRNA in DENV-2-infected cells and interacted with the DENV-2 NS3 (protease/helicase) protein, suggesting a possible interaction of viperin at sites of DENV-2 replication^24^. In addition, viperin can also restrict TBEV RNA replication and it would be logical to assume that common molecular mechanisms are at play. However this is not the case as restriction of TBEV is dependent on a radical S-adenosyl-L-methionine (SAM) domain within viperin while this domain is dispensable for DENV and HCV restriction^28^. Interestingly, the carboxy-terminus of viperin is important for restriction of DENV while for HCV restriction the carboxy-terminus and the amino-terminal amphipathic helix are important with the latter crucial for viperin’s interaction with NS5A and VAP-A^24, 25^. To this end we show using a panel of viperin mutants that the antiviral activity of viperin is similar to that of DENV in that the carboxy-terminus of viperin is essential while the amino–terminus and the SAM domain are dispensable. This is not surprising given the similarity between DENV and ZIKV and suggests that like DENV viperin interferes with ZIKV RNA replication and future studies are required to determine if viperin interacts with ZIKV replication complex proteins and/or essential pro-viral host factors. Understanding how viperin restricts ZIKV is not only important to our understanding of the innate host response to infection but also in our understanding of the ZIKV life cycle. Ultimately this knowledge may be used to develop therapeutic options to combat emerging viral infections.

## Materials and Methods

### Cells and Culture Conditions

All mammalian cell lines were maintained at 37 °C in a 5% CO_2_ air atmosphere. The human hepatoma cell line Huh-7 was maintained as previously described^25^. The placental lines, HTR8/SVneo cells (from Professor Charles Graham, Queen’s University, Kingston, Ontario) and JEG3 cells (purchased from ATCC) were maintained in RPMI-1640 media supplemented with 10% FBS, penicillin and streptomycin (P/S). Vero cells were cultured in DMEM supplemented with 10% FBS and P/S. Murine Embryonic Fibroblasts (MEFs) were prepared from day 13.5–14.5 embryos. Isolated MEFs were maintained in DMEM supplemented with 10% FBS and P/S. Neural progenitor cells (NPCs) were isolated from E14-18 embryonic cortices and grown as neurospheres as previously described^47^. Cells were grown in the presence of growth factors Epidermal Growth Factor (rhEGF, 20 ng/ml; STEMCELL Technologies) and Fibroblast Growth Factor (bFGF2, 10 ng/ml; GIBCO). NPCs from dissociated neurospheres (at first or second passage) were plated onto poly-l-lysine coated coverslips and culture surfaces at a density of 50,000 cells/cm^2^, and cultured overnight prior to infection with ZIKV, as indicated. C6/36 *Aedes albopictus* cells were maintained in Basal Medium Eagle (BME) supplemented with L-glutamine, MEM non-essential amino acids, sodium pyruvate, 10% FBS and P/S and cultured at 28 °C with 5% CO_2_. Human monocyte-derived macrophages (MDM) were prepared by adherence of PBMC isolated from blood from healthy donors provided by the Australian Red Cross Blood Service and were cultured for 5 days in the presence of 7.5% (v/v) human serum, as described previously^48^.

### Viruses

The ZIKV strains MR766 (Uganda, 1947) and PRVABC59 (Puerto Rico, 2015) were propagated in C6/36 cells by infection at a multiplicity of infection (MOI) of 0.01 and supernatants harvested at 5–6 days post-infection. PRVABC59 is a contemporary strain that was isolated by CDC from the serum of a ZIKV infected patient who travelled to Puerto Rico in 2015. The complete genome sequence is published (Ref. Gene bank accession # KU501215). DENV infections utilised, Mon601, a derivative of the New Guinea C strain that was produced from *in vitro* transcribed RNA, transfected into BHK-21 baby hamster kidney cells, amplified in C6/36 insect cells and titered in Vero cells.

### Plaque Assay

Virus infectivity was determined by plaque assay. Briefly, Vero cells in 6-well trays at approximately 90% confluency were infected with 800 µl of serially-diluted virus-containing supernatants for 1 hour at 37 °C. Supernatants were then replaced with a 2ml overlay of complete media containing 1.5% (w/v) carboxymethylcellulose (CMC) (Sigma) and cells returned to culture for 5–6 days. Cell monolayers were then fixed by addition of 2 ml of 10% formalin and incubation for 1 h. The CMC overlay was then removed and plaques were visualised via crystal violet stain. Plaques were counted and virus infectivity expressed as plaque-forming units (PFU) per ml.

### Plasmids and transfections

The human viperin cDNA expression plasmid with an N-terminal FLAG tag in the pLenti6/V5-D-TOPO plasmid was previously described^24^. Transfection of plasmids and PolyI:C (Sigma) was performed using Lipofectamine 3000 (Thermo Fisher Scientific) according to the manufacturer’s recommendations. Plasmids expressing a constitutively active RIG-I (RIG-N), the IFN-β promoter driving luciferase and viperin WT and mutant plasmids have been described elsewhere^24, 49^.

### Real-time PCR

Extraction of total cellular RNA, first-strand cDNA synthesis and real-time qRT-PCR was performed as described previously^26^. All primer sequences used are outlined in Supplementary Table 1. Primer sequences for ZIKV RNA have been described previously^50^. Generation of a ZIKV RT-PCR standard curve used plasmids pFK-DVs (DENV-2, strain 16681)^51^ and pZIKV-ICD (strain Paraiba_01/2015)^52^.

### Antibodies

Mouse anti-envelope glycoprotein 4G2 (D1-4G2-4-15) was prepared from hybridoma cells purchased from ATCC. Mouse anti-dsRNA 3G1 was a generous gift from Roy Hall (University of Queensland)^53^. Mouse anti-viperin (ab107359), rabbit anti-PAX6 (ab2237) and rabbit anti-phospho(S10) Histone H3 (ab5176) were obtained from Abcam. Rabbit anti-SOX2 (ab5603) was obtained from Merck Millipore. Rabbit anti-GFAP (G9269), rabbit anti-β-tubulin isotype III (T2200) and mouse anti-β-actin (AC15) was purchased from Sigma-Aldrich. Mouse anti-NS4B mAb 44-4-7 was generously provided by Qing Yin Wang (Novartis Institute for Tropical Diseases, Singapore). All secondary antibodies were purchased from Thermo Fisher Scientific.

### Immunofluorescence Microscopy

Immunofluorescent labelling and wide-field fluorescence microscopy was performed essentially as described^54^. Briefly, cells growing in cell culture plates or on glass coverslips coated with poly-L-lysine (0.1% [w/v]) were washed with PBS, fixed with 4% paraformaldehyde in PBS for 15 min at room temperature and permeabilised with 0.1% Triton X-100 in PBS for 15 min at 4 °C. Alternatively, cells were fixed with ice-cold acetone:methanol (1:1) for 5 minutes at 4 °C. Samples were then blocked with 5% BSA in PBS for 30 min at room temperature and incubated with primary antibody diluted in PBS/1% BSA for 1 h at room temperature. After washing twice with PBS, cells were incubated with Alexa Fluor-conjugated secondary antibody diluted 1:200 in PBS/1%BSA for 1 h at 4 °C in the dark. Samples were then washed with PBS and incubated with DAPI (Sigma-Aldrich, 1 µg/ml) for 15 min at room temperature. Samples were then washed with PBS and, for cells grown on coverslips, mounted with Vectashield Antifade Mounting Medium (Vector Laboratories). Images were then acquired using a Nikon TiE inverted fluorescent microscope equipped with Plan Apochromat 60× NA 1.4 oil immersion, Super Plan Fluor 40× NA 0.4, Super Plan Fluor 20× NA 0.4 and Plan Fluor 10× NA 0.3 objectives (Nikon). Illumination was provide by an Intensilight C-HGFIE Precentered Fiber Illuminator mercury light source (Nikon), while BrightLine single-band filter sets (DAPI-5060C-NTE-ZERO, FITC-3540C-NTE-ZERO and TxRed-4040C-NTE-ZERO) were from Semrock and emitted light was collected with a monochrome 12-bit cooled charge-coupled device (CCD) camera with a maximum resolution of 1,280 × 1,024 (DS-Qi1; Nikon). Unless otherwise indicated images were processed using NIS Elements AR v.3.22. (Nikon) and Photoshop 6.0 (Adobe) software. In most instances contrast stretching was applied using the ‘Autoscale’ function of NIS Elements v.3.22.

### Immunoblotting

Western blotting was performed essentially as described elsewhere^55^. Membrane bound protein was detected by chemiluminescence using SuperSignal West Femto (Pierce) and imaged using a ChemiDoc MP imaging system (Bio-Rad).

### Generation of Viperin null mice

A CRISPR gRNA was designed to target exon 1 of murine Viperin (5′-agggtggctagatcccggga-3′) using CRISPR Design tool (crispr.mit.edu) and then generated according to the protocol as previously described^56^. gRNA IVT was performed using HiScribe™ T7 Quick High Yield RNA Synthesis Kit. Cas9 mRNA was generated by IVT using mMESSAGE mMACHINE® T7 ULTRA Transcription Kit (Ambion) from pCMV/T7-hCas9 (Toolgen) digested with *Xho*I. gRNAs and Cas9 mRNA were purified using MEGAclear™ Transcription Clean-Up Kit (Ambion). CRISPR gRNA (50ng/mL) and gRNA pairs (100 ng/mL each) were injected into C57BL/6N zygotes, transferred to psuedopregnant recipients and allowed to develop to term as previously described^57^. Founder pups were screened for exon deletion by PCR amplification across the targeted region (F-5′gtgtttgcctggaatataccagtcttgagtcct -3′, R-5′ - gacaatctgcaaggattgaatgcta -3′). PCR products from founders with deletions were Sanger sequenced to identify specific mutations. Routine genotyping was performed by PCR with the above primers spanning the target region and separated on 1% agarose gels. The University of Adelaide animal ethics committee approved generation and experiments involving viperin null mice.

### Statistics

Data were graphed and analysed within the Prism 7 software (Graphpad Software Inc CA) using either T tests or Ordinary One or Two way Anova, all tests were corrected for multiple comparison using the Holm-Sidak method.

## Electronic supplementary material

Supplementary Figures Combined

## References

[1] Lazear HM, Diamond MS (2016). Zika virus: new clinical syndromes and its emergence in the Western Hemisphere. Journal of virology.

[2] Duffy MR (2009). Zika virus outbreak on Yap Island, Federated States of Micronesia. N Engl J Med.

[3] Rasmussen SA, Jamieson DJ, Honein MA, Petersen LR (2016). Zika Virus and Birth Defects–Reviewing the Evidence for Causality. N Engl J Med.

[4] Driggers RW (2016). Zika virus infection with prolonged maternal viremia and fetal brain abnormalities. New England Journal of Medicine.

[5] Quicke KM (2016). Zika Virus Infects Human Placental Macrophages. Cell Host Microbe.

[6] Tabata T (2016). Zika Virus Targets Different Primary Human Placental Cells, Suggesting Two Routes for Vertical Transmission. Cell Host Microbe.

[7] Lazear HM (2016). A Mouse Model of Zika Virus Pathogenesis. Cell Host Microbe.

[8] Li C (2016). Zika Virus Disrupts Neural Progenitor Development and Leads to Microcephaly in Mice. Cell Stem Cell.

[9] Li H (2016). Zika Virus Infects Neural Progenitors in the Adult Mouse Brain and Alters Proliferation. Cell Stem Cell.

[10] Miner JJ (2016). Zika Virus Infection during Pregnancy in Mice Causes Placental Damage and Fetal Demise. Cell.

[11] D’Ortenzio E (2016). Evidence of Sexual Transmission of Zika Virus. N Engl J Med.

[12] Musso D (2015). Potential sexual transmission of Zika virus. Emerg Infect Dis.

[13] Tang WW (2016). A Mouse Model of Zika Virus Sexual Transmission and Vaginal Viral Replication. Cell Reports.

[14] Wilkins C, Gale M (2010). Recognition of viruses by cytoplasmic sensors. Curr Opin Immunol.

[15] Jensen S, Thomsen AR (2012). Sensing of RNA viruses: a review of innate immune receptors involved in recognizing RNA virus invasion. J Virol.

[16] Beachboard DC, Horner SM (2016). Innate immune evasion strategies of DNA and RNA viruses. Curr Opin Microbiol.

[17] Gack MU, Diamond MS (2016). Innate immune escape by Dengue and West Nile viruses. Curr Opin Virol.

[18] Grant A (2016). Zika Virus Targets Human STAT2 to Inhibit Type I Interferon Signaling. Cell Host Microbe.

[19] Kumar A (2016). Zika virus inhibits type-I interferon production and downstream signaling. EMBO Rep.

[20] Aliota MT (2016). Characterization of Lethal Zika Virus Infection in AG129 Mice. PLoS Negl Trop Dis.

[21] Lazear HM (2016). A mouse model of Zika virus pathogenesis. Cell host & microbe.

[22] Yockey LJ (2016). Vaginal Exposure to Zika Virus during Pregnancy Leads to Fetal Brain Infection. Cell.

[23] Helbig KJ, Beard MR (2014). The role of viperin in the innate antiviral response. J Mol Biol.

[24] Helbig KJ (2013). Viperin is induced following dengue virus type-2 (DENV-2) infection and has anti-viral actions requiring the C-terminal end of viperin. PLoS Negl Trop Dis.

[25] Helbig KJ (2011). The antiviral protein viperin inhibits hepatitis C virus replication via interaction with nonstructural protein 5A. Hepatology.

[26] Helbig KJ, Lau DT, Semendric L, Harley HA, Beard MR (2005). Analysis of ISG expression in chronic hepatitis C identifies viperin as a potential antiviral effector. Hepatology.

[27] Szretter KJ (2011). The interferon-inducible gene viperin restricts West Nile virus pathogenesis. J Virol.

[28] Upadhyay AS (2014). Viperin is an iron-sulfur protein that inhibits genome synthesis of tick-borne encephalitis virus via radical SAM domain activity. Cell Microbiol.

[29] Wang S (2012). Viperin inhibits hepatitis C virus replication by interfering with binding of NS5A to host protein hVAP-33. J Gen Virol.

[30] Hanners NW (2016). Western Zika Virus in Human Fetal Neural Progenitors Persists Long Term with Partial Cytopathic and Limited Immunogenic Effects. Cell Rep.

[31] Graham CH (1993). Establishment and characterization of first trimester human trophoblast cells with extended lifespan. Exp Cell Res.

[32] Garcez PP (2016). Zika virus impairs growth in human neurospheres and brain organoids. Science.

[33] Li, C. *et al*. Zika virus disrupts neural progenitor development and leads to microcephaly in mice. *Cell stem cell* (2016). 10.1016/j.stem.2016.04.017 27179424

[34] Tang H (2016). Zika Virus Infects Human Cortical Neural Progenitors and Attenuates Their Growth. Cell Stem Cell.

[35] Teng TS (2012). Viperin restricts chikungunya virus replication and pathology. J Clin Invest.

[36] Schneider WM, Chevillotte MD, Rice CM (2014). Interferon-stimulated genes: a complex web of host defenses. Annu Rev Immunol.

[37] Miner JJ (2016). Zika virus infection during pregnancy in mice causes placental damage and fetal demise. Cell.

[38] Grandvaux N (2002). Transcriptional profiling of interferon regulatory factor 3 target genes: direct involvement in the regulation of interferon-stimulated genes. J Virol.

[39] Wathelet MG (1998). Virus infection induces the assembly of coordinately activated transcription factors on the IFN-beta enhancer *in vivo*. Mol Cell.

[40] Aguirre S (2012). DENV inhibits type I IFN production in infected cells by cleaving human STING. PLoS Pathog.

[41] Anglero-Rodriguez YI, Pantoja P, Sariol CA (2014). Dengue virus subverts the interferon induction pathway via NS2B/3 protease-IkappaB kinase epsilon interaction. Clin Vaccine Immunol.

[42] Dalrymple NA, Cimica V, Mackow ER (2015). Dengue Virus NS Proteins Inhibit RIG-I/MAVS Signaling by Blocking TBK1/IRF3 Phosphorylation: Dengue Virus Serotype 1 NS4A Is a Unique Interferon-Regulating Virulence Determinant. MBio.

[43] Narayana SK (2015). The Interferon-induced Transmembrane Proteins, IFITM1, IFITM2, and IFITM3 Inhibit Hepatitis C Virus Entry. J Biol Chem.

[44] Savidis G (2016). The IFITMs Inhibit Zika Virus Replication. Cell Rep.

[45] Nasr N (2012). HIV-1 infection of human macrophages directly induces viperin which inhibits viral production. Blood.

[46] Wang X, Hinson ER, Cresswell P (2007). The interferon-inducible protein viperin inhibits influenza virus release by perturbing lipid rafts. Cell Host Microbe.

[47] Jolly LA, Homan CC, Jacob R, Barry S, Gecz J (2013). The UPF3B gene, implicated in intellectual disability, autism, ADHD and childhood onset schizophrenia regulates neural progenitor cell behaviour and neuronal outgrowth. Hum Mol Genet.

[48] Wati S, Li P, Burrell CJ, Carr JM (2007). Dengue virus (DV) replication in monocyte-derived macrophages is not affected by tumor necrosis factor alpha (TNF-alpha), and DV infection induces altered responsiveness to TNF-alpha stimulation. J Virol.

[49] Helbig KJ (2009). Differential expression of the CXCR3 ligands in chronic hepatitis C virus (HCV) infection and their modulation by HCV *in vitro*. J Virol.

[50] Waggoner JJ (2016). Single-Reaction Multiplex Reverse Transcription PCR for Detection of Zika, Chikungunya, and Dengue Viruses. Emerging infectious diseases.

[51] Fischl W, Bartenschlager R (2013). High-throughput screening using dengue virus reporter genomes. Methods Mol Biol.

[52] Tsetsarkin, K. A. *et al*. A Full-Length Infectious cDNA Clone of Zika Virus from the 2015 Epidemic in Brazil as a Genetic Platform for Studies of Virus-Host Interactions and Vaccine Development. *MBio* **7**, doi:10.1128/mBio.01114-16 (2016). 10.1128/mBio.01114-16 PMC4999549 27555311

[53] O’Brien CA (2015). Viral RNA intermediates as targets for detection and discovery of novel and emerging mosquito-borne viruses. PLoS neglected tropical diseases.

[54] Eyre, N. S. *et al*. Phosphorylation of NS5A Serine-235 is essential to hepatitis C virus RNA replication and normal replication compartment formation. *Virology* **491**, 27–44, doi:10.1016/j.virol.2016.01.018 (2016) 10.1016/j.virol.2016.01.018 26874015

[55] Eyre NS, Drummer HE, Beard MR (2010). The SR-BI partner PDZK1 facilitates hepatitis C virus entry. PLoS Pathog.

[56] Cong L (2013). Multiplex genome engineering using CRISPR/Cas systems. Science.

[57] Yang H (2013). One-step generation of mice carrying reporter and conditional alleles by CRISPR/Cas-mediated genome engineering. Cell.

