# Supplementary material for: Viperin is an important host restriction factor in control of Zika virus infection
Source: Sci Rep. 2017 Jun 30;7:4475. doi: 10.1038/s41598-017-04138-1 (PMC5493656; doi:10.1038/s41598-017-04138-1)
Supplement: Supplementary file 1 — Supplementary Figures Combined [file 41598_2017_4138_MOESM1_ESM.pdf]

Supplementary Information for:

Viperin is an important host restriction factor in control of Zika virus infection

Authors: *Kylie, H Van der Hoek,[1,3, †] Nicholas S Eyre,[1,3, †] Byron Shue,[1,3, †] Onruedee Khantisitthiporn, [1,3] Kittirat Glab-Ampi,[1,3] Jillian M Carr,[4] Matthew J Gartner,[1,3] Lachlan A Jolly,[2] Paul Q Thomas,[1] Fatwa Adikusuma,[1] Tanja Jankovic-Karasoulos,[2] Claire T Roberts,[2] Karla J Helbig, and Michael R Beard[1,3,*]*

Supplementary Table S1: Oligonucleotide Primers for qRT-PCR

Target	Species	Direction	Sequence (5' → 3')
Viperin	Human	For	AATTGAATTCATGTGGGTGCTTACACCTGCTG
Viperin	Human	Rev	AATAGGATCCCTACCAATCCAGCTTCAGATCA
IFIT1	Human	For	AACTTAATGCAGGAAGAACATGACAA
IFIT1	Human	Rev	CTGCCAGTCTGCCCATGTG
IFN-β	Human	For	TGTCAACATGACCAACAAGTGTCT
IFN-β	Human	Rev	GCAAGTTGTAGCTCATGGAAAGAG
IFITM1	Human	For	CGCCAAGTGCCTGAACATCT
IFITM1	Human	Rev	CCCGTTTTTCCTGTATTATCTGTA
ISG15	Human	For	TGGCGGGCAACGAATT
ISG15	Human	Rev	GGGTGATCTGCGCCTTCA
OAS1	Human	For	TCCACCTGCTTCACAGAACTACA
OAS1	Human	Rev	GGCGGATGAGGCTCTTGAG
MX1	Human	For	CAGCACCTGATGGCCTATCAC
MX1	Human	Rev	CATGAAGAACTGGATGATCAAAGG
RPLPO / 36B4	H / M	For	AGATGCAGCAGATCCGCAT
RPLPO / 36B4	H / M	Rev	GGATGGCCTTGCGCA
Viperin	Mouse	For	TTGGGCAAGCTTGTGAGATTC
Viperin	Mouse	Rev	TGAACCATCTCTCCTGGATAAGG
IFIT1	Mouse	For	TCGCGTAGACAAAGCTCTTCATC
IFIT1	Mouse	Rev	AGCAGAGCCCTTTTTGATAATGTAA
IFN-β	Mouse	For	AGAAAGGACGAACATTCGGAAA
IFN-β	Mouse	Rev	CCGTCATCTCCATAGGGATCTT
MxA	Mouse	For	TGCCTGGCAGAGAGACTGACT
MxA	Mouse	Rev	GCTTGCACTCTGATGACTGCTATT

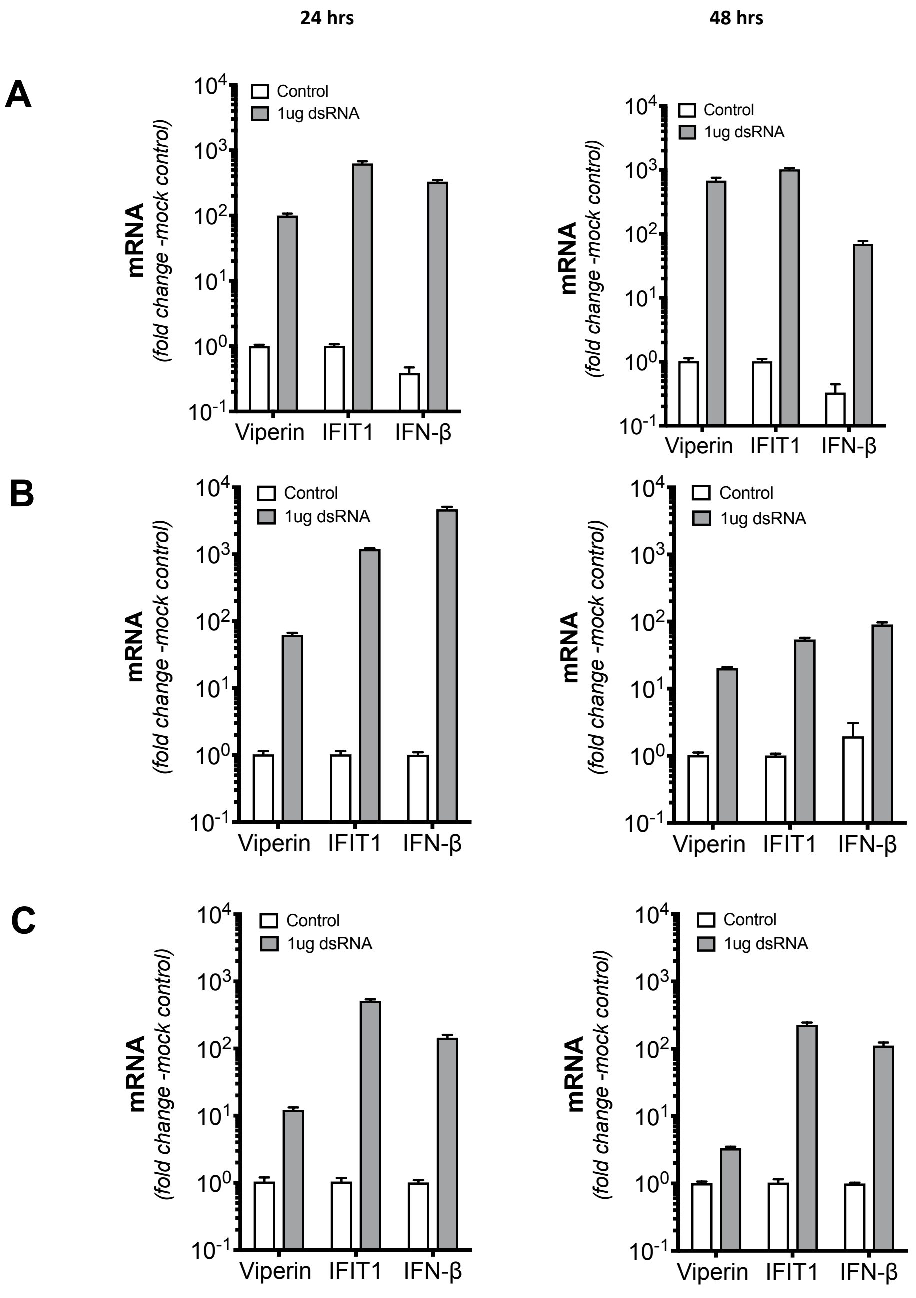

Supplementary Fig S1. Huh-7, HTR8/SVNeo and Jeg3 cells are innate immune competent. Huh-7 **(A)**, HTR8/SVNeo **(B)** and Jeg3 **(C)** cells were transfected with poly I:C (1µg) using DIMRIE-C and ISG expression was quantified by qRT-PCR 24 hrs post stimulation. Data are normalised to the RPLPO housekeeping gene and expressed as a fold-change relative to mock-transfected control (data are means + SD, n=3).

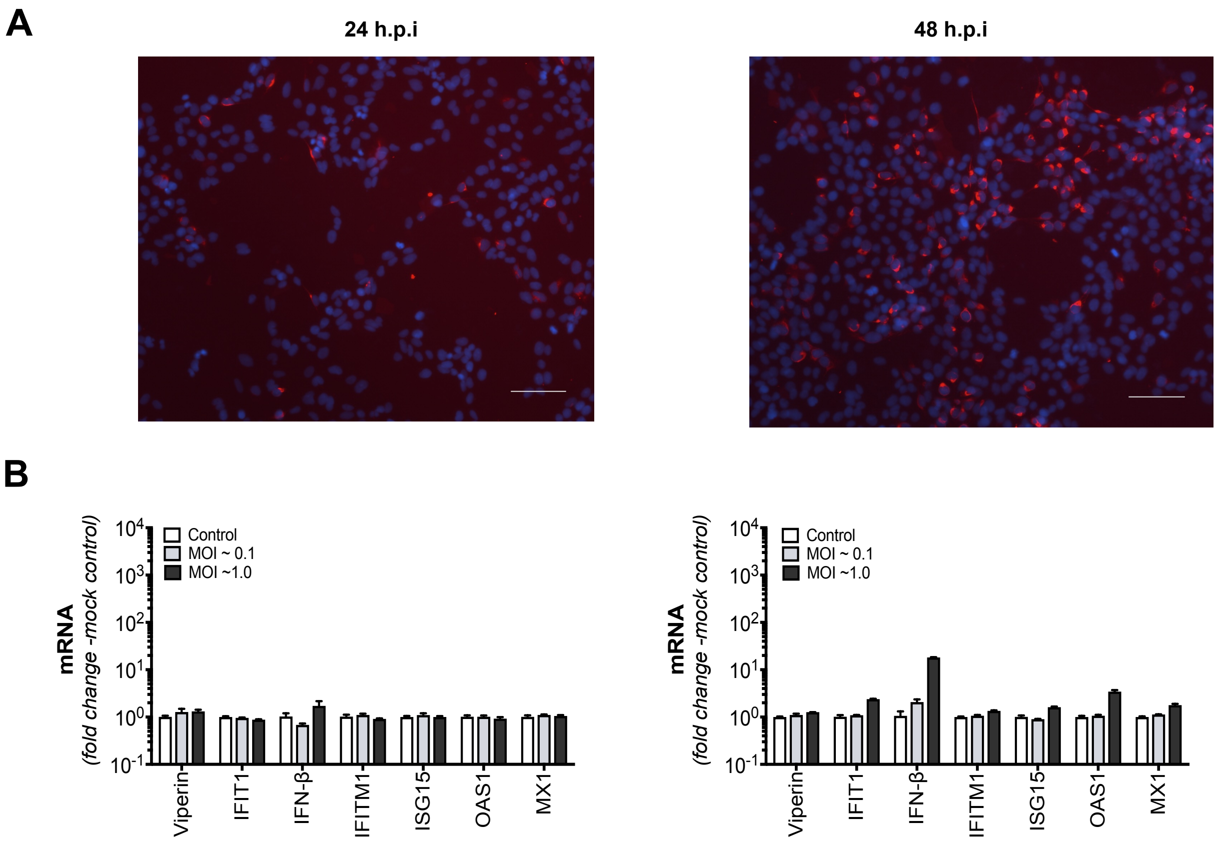

Supplementary Fig S2. ZIKV strain PRVABC59 infection results in an attenuated ISG response in HTR8/SVNeo cells. HTR8/SVNeo cells were infected with ZIKV (PRVABC59) at indicated multiplicity of infection (MOI). **(A)** Indirect immunofluorescence of ZIKV infection at 24 and 48 h.p.i at MOI 1. Cells were stained with the 4G2 antibody to detect ZIKV E antigen (red) and DAPI DNA stain (blue). Scale bars represent 100 μm. **(B)** At 24 and 48 h.p.i total RNA was extracted and qRT-PCR was used to detect mRNA for IFN-β and indicated ISGs. Data are normalised to the RPLPO housekeeping gene and expressed as a fold-change relative to mock-infected control (data are means + SD, n=3).

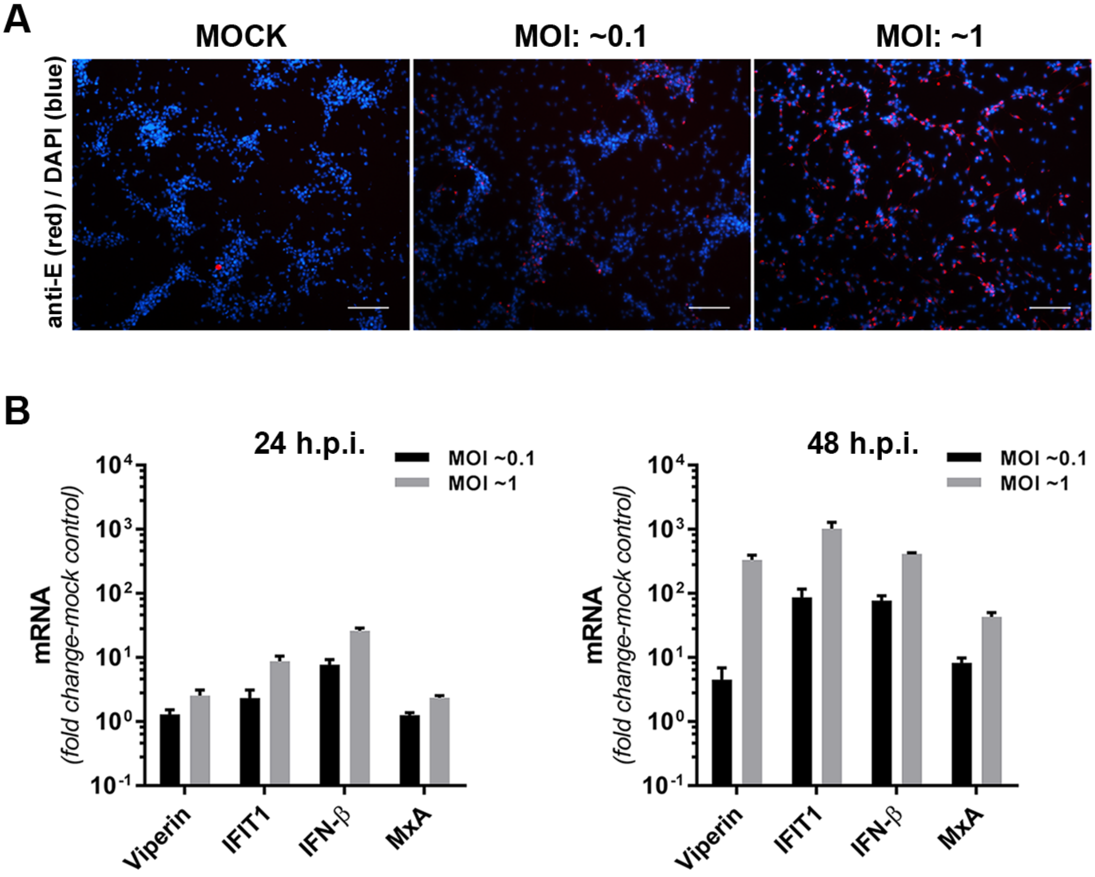

Supplementary Fig S3. ZIKV strain PRVABC59 infection results in an attenuated ISG response in neural progenitor cells (NPC). NPCs were infected with ZIKV (PRVABC59) at indicated multiplicity of infection (MOI). **(A)** Indirect immunofluorescence of ZIKV infection at 48 h.p.i at MOI 1. Cells were stained with the 4G2 antibody to detect ZIKV E antigen (red) and DAPI DNA stain (blue). Scale bars represent 20 µm. **(B)** At 24 and 48 h.p.i total RNA was extracted and qRT-PCR was used to detect mRNA for IFN-β and indicated ISGs. Data are normalised to the RPLPO housekeeping gene and expressed as a fold-change relative to mock-infected control (data are means + SD, n=3).

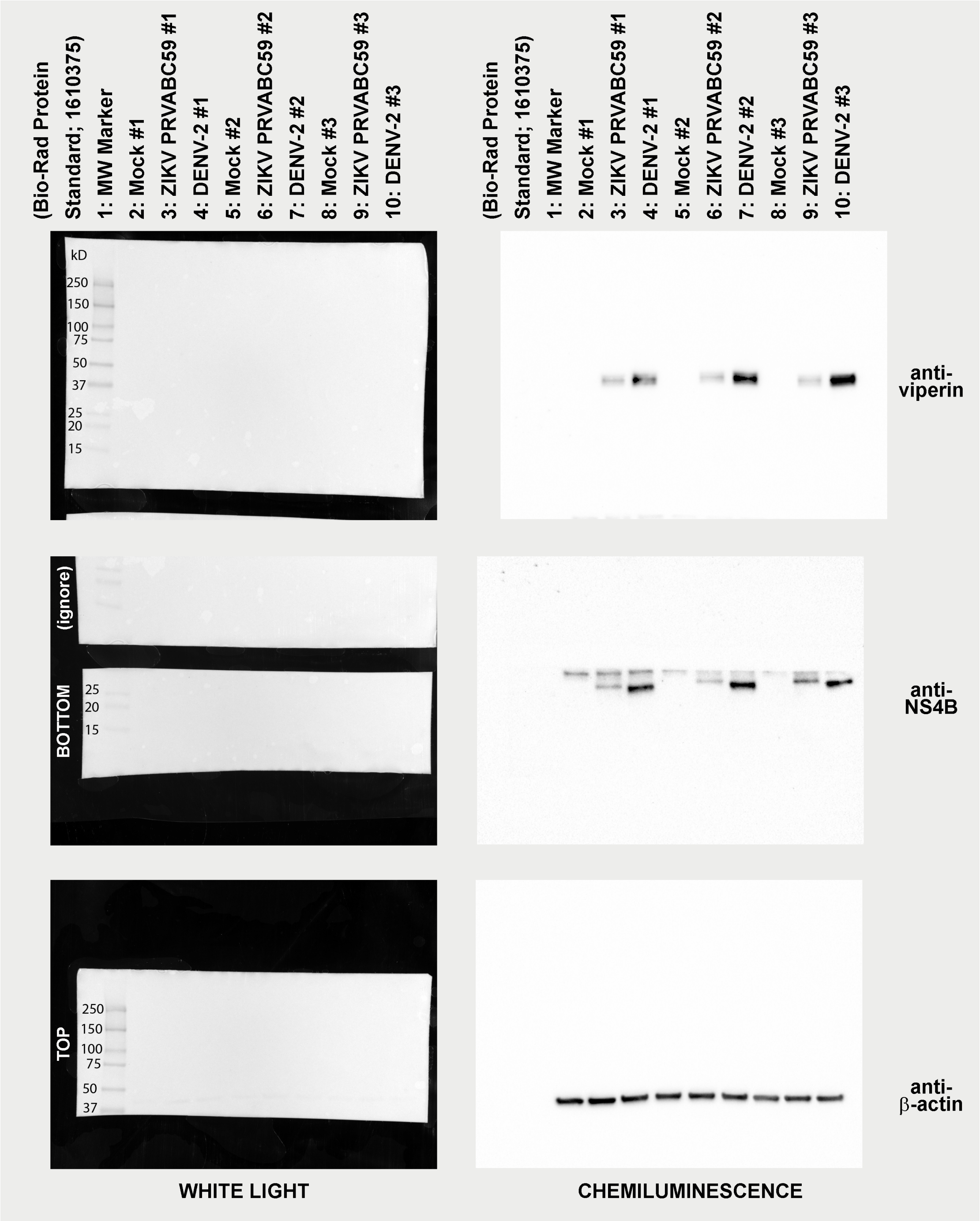

Supplementary Fig S4. Unprocessed immunoblots that relate to figure 4.

(Bio-Rad Protein Standard; 1610375)
1: MW Marker
2: Viperin WT ZIKV (-)
3: Viperin KO ZIKV (-)
4: Viperin WT MR766
5: Viperin WT PRVABC59
6: Viperin KO MR766
7: Viperin KO PRVAC59

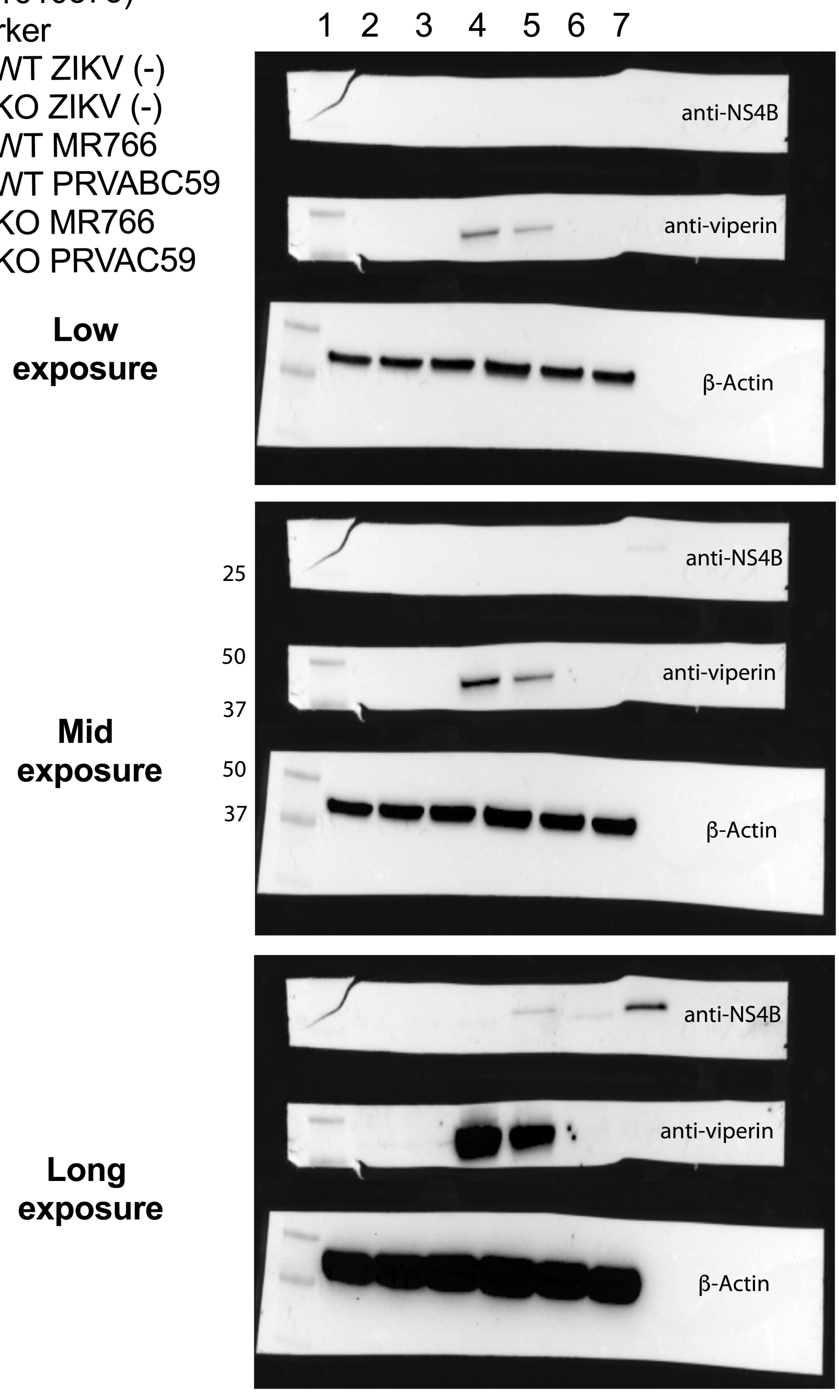

Supplementary Fig S5. Unprocessed immunoblots that relate to figure 7.
